# Stochastic Vibrations of a System of Plates Immersed in Fluid Using a Coupled Boundary Element, Finite Element, and Finite Difference Methods Approach

**DOI:** 10.3390/ma16093583

**Published:** 2023-05-07

**Authors:** Michał Guminiak, Marcin Kamiński, Agnieszka Lenartowicz, Maciej Przychodzki

**Affiliations:** 1 Institute of Structural Analysis, Poznan University of Technology, Piotrowo 5 Street, 60-965 Poznan, Poland; 2 Department of Structural Mechanics, Faculty of Civil Engineering, Architecture and Environmental Engineering, Łódź University of Technology, Al. Politechniki 6, 90-924 Łódź, Poland; 3 Doctoral School, Poznan University of Technology, Piotrowo 3 Street, 60-965 Poznan, Poland

**Keywords:** natural vibrations of thin plates in fluid, numerical method, stochastic perturbation method, Monte Carlo simulation, semi-analytical probabilistic approach

## Abstract

The main objective of this work is to investigate the natural vibrations of a system of two thin (Kirchhoff–Love) plates surrounded by liquid in terms of the coupled Stochastic Boundary Element Method (SBEM), Stochastic Finite Element Method (SFEM), and Stochastic Finite Difference Method (SFDM) implemented using three different probabilistic approaches. The BEM, FEM, and FDM were used equally to describe plate deformation, and the BEM was applied to describe the dynamic interaction of water on a plate surface. The plate’s inertial forces were expressed using a diagonal or consistent mass matrix. The inertial forces of water were described using the mass matrix, which was fully populated and derived using the theory of double-layer potential. The main aspect of this work is the simultaneous application of the BEM, FEM, and FDM to describe and model the problem of natural vibrations in a coupled problem in solid–liquid mechanics. The second very important novelty of this work is the application of the Bhattacharyya relative entropy apparatus to test the safety of such a system in terms of potential resonance. The presented concept is part of a solution to engineering problems in the field of structure and fluid dynamics and can also be successfully applied to the analysis of the dynamics of the control surfaces of ships or aircraft.

## 1. Introduction

One of the computational numerical tools is the Boundary Element Method (BEM), which may be applied in the numerical analysis of a variety of engineering and scientific problems, e.g., thin or thick plate bending. The mathematical foundation of this method began with the theory of potential developed by Kellog [1]. Following, Kupradze [2] and Michlin [3] proposed multidimensional and singular boundary integral equations for selected parts of the theory of elasticity. Further works were carried out by Jaswon [4], Symm [5], Jaswon, and Ponter [6], who proposed a direct application of the integral equations to static potential problems. The above investigation can be treated as the theoretical foundations of the boundary integral equation method. The BEM is an independent tool that may be optimal for selected engineering problems. The main advantage of the BEM approach is its simplicity in formulating a large number of structural mechanics problems based on the theories of potential or elasticity. For some problems, this method is irreplaceable. A significant degree of discretization is not required using the BEM approach. The problem of plate bending was solved using the BEM by Stern [7], Altiero and Sikarskie [8], and Bèzine and Gamby [9]. The effectiveness of the BEM and FEM was compared by Debbich [10,11] for the stress analysis of plates of medium thickness. A modification of the Kirchhoff–Love plate theory in terms of the BEM was performed by El-Zafrany, Debbih, and Fadhil [12], and Burczyński [13] comprehensively described the application of the BEM to various solid mechanics problems. The dynamics of the plates were discussed by Beskos and Wen [14] as well as Aliabadi and Young [15]. Katsikadelis [16], Katsikadelis and Sapountzakis [17], and Katsikadelis and Yotis [18] developed many contributions on plate analysis. Particularly noteworthy is the work of Katsikadelis and Babouskos [19], in which the authors presented a nonlinear flutter instability problem of thin damped plates in BEM terms using the Analog Equation Method (AEM), of which the foundations were developed by Katsikadelis [20]. In the field of plate bending analysis, Guminiak, Okupniak, and Sygulski [21] proposed a modified and simplified formulation of the boundary integral equations for the Kirchhoff–Love plate bending problem. This concept was extended to the static, dynamic, and stability problems of a thin plate, together with a number of numerical examples, e.g., Guminiak [22,23]. Kamiński presented a number of works describing the probabilistic techniques of the BEM, particularly in the context of the stochastic perturbation approach, e.g., [24,25], whereas Łasecka-Plura and Lewandowski [26] presented the method of the frequency response function development for frame with dampers considering uncertain design parameters.

Probabilistic approaches play a fundamental role in the investigations presented here. Material constants, structural geometrical parameters, and also some other quantities in the presented analyses form a set of variables defined as continuous or discrete random variables. Their estimations are typically performed using the stochastic perturbation technique (SPT), semi-analytical method (SAM), and Monte Carlo simulation (MCS). In this field, a very useful probabilistic approach is the stochastic spline fictitious BEM applied with reliability analyses for planar elasticity [27] as well as Reissner plate bending [28]. This method seems to be highly efficient in fracture mechanics for the analysis of the presence of cracks [29] as well as stress intensity factors [30]. The Stochastic Boundary Element Method (SBEM) was newly proposed for investigations of fracture and fatigue behavior [31] and analysis of tunnels [32] and dams [33]. A coupled Finite and Boundary Element Method linked with the least squares point interpolation approach coupled with the stochastic perturbation method for a structure-acoustic system have been investigated by Zang et al. [34]. Another research branch is the stochastic extension of the scaled Boundary Finite Element Method [35,36,37] provided according to the Karhunen–Loeve expansion and the Monte Carlo simulation (MCS). Comparatively, the SPT technique allows for an approximate solution to the boundary value problem that cannot be accurately treated [38,39]. The simplest stochastic approach seems to be the semi-analytical method (SAM). This technique appears to be highly efficient. It is based on the numerical approximation of the structural response using polynomial bases obtained by a finite number of deterministic solutions (playing the role of a series of numerical tests) and the Least Squares Method (LSM) approximation [40].

The dynamic analysis of fluid–structure interactions is a subject of many papers abd books in the available literature. One of the most important elements of such an analysis is the derivation of the fluid mass matrix. The proposition of the fluid mass matrix derivation is given in detail below; however, a fluid–structure interaction problem was included in many studies, e.g., [41,42,43]. This problem was also investigated analytically by Liang et al. [44]. The natural vibrations of a system of two plates submerged in an incompressible and inviscid fluid were investigated by Guminiak and Sygulski using the BEM-BEM approach [45].

This work presents a free vibration analysis of a system of two rectangular thin (Kirchhoff–Love) plates with constant thickness that are totally immersed in a fluid (water). It is assumed that the fluid is an inviscid and incompressible medium coupled with a plate. The fluid causes no separate flow resulting from the plate vibration. The presence of the fluid was described using a nonsymmetric and fully populated additional mass matrix, which was adjoined (coupled) to the plate mass matrix. Generally, the mass matrix of a fluid is derived using the potential theory and the direct BEM approach presented by Sygulski [46,47]. This approach, coupled with BEM, FEM, and FDM descriptions of plate deformation, was applied by Lenartowicz et al. [48] to determine the free vibration of iso- and orthotropic plates fully submerged in a fluid. The semi-analytical stochastic boundary element analysis of a single plate fully submerged in a fluid was investigated by Guminiak and Kamiński [49]. It is worth noting that fluid–structure interaction problems have been comprehensively described by Morand and Ohayon [50].

In the present work, three coupled approaches will be introduced: BEM-BEM, FEM-BEM, and FDM-BEM, where the presence of the fluid is always determined by the BEM, and plate deformation will be described by the BEM, FEM, and FDM, respectively. The response of the system of two plates submerged in a fluid medium is described by the natural frequencies, which are the basis for probabilistic analysis. The first important aspect of this work is the simultaneous application of the BEM, FEM, and FDM to describe and model the problem of natural vibrations in a coupled problem in solid–liquid mechanics. The second equally important aspect is the use of a relative entropy apparatus to test the safety of such a system in terms of potential resonance.

The current work aims to present a simple and effective concept for solving the problem of the interaction of a system of plates surrounded by a liquid. The presented concept can be successfully applied in the engineering analysis of the dynamics of ships or aircraft control surfaces.

## 2. Fluid–Structure Inertial Interaction for a Single Plate

It was assumed that the considered fluid is incompressible and inviscid. There was no separate flow between the plate and fluid, and no fluid moved along the plane direction of the plate. The surrounding fluid is the source of the additional inertial forces.

It was assumed that the velocity potential of a fluid for small disturbances is expressed as

(1) $$V\left(x,t\right)=\tilde{V}\left(x\right)\cdot e^{i\omega t}$$

where *t* represents time, $x=\left(x,y,z\right)$ is the position vector of a point P arbitrarily assumed in space, and *ω* expresses the circular frequency of a fluid. It was assumed that the velocity potential of liquid (1) must satisfy the Laplace equation.

(2) $$\nabla^{2}\tilde{V}=0$$

**Figure 1:**
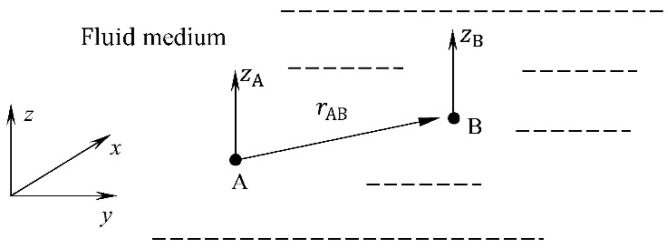
Determination of the fluid velocity potential.

The solution of Equation (2) can be expressed as the double-layer potential in terms of the boundary integral equation.

(3) $$\tilde{V}\left(P\right)=\int_{S}\left({\tilde{V}}_{1}\left(Q\right)-{\tilde{V}}_{2}\left(Q\right)\right)\frac{\partial V^{*}\left(P,Q\right)}{\partial z_{Q}}dS_{Q},$$

wherein

(4) $$V^{*}\left(P,Q\right)=\frac{1}{4\pi }\cdot \frac{1}{r\left(P,Q\right)}$$

is the fundamental solution of the Laplace Equation (2), $i=\sqrt{-1}$, ${\tilde{V}}_{1}\left(Q\right)$ and ${\tilde{V}}_{2}\left(Q\right)$ are the amplitudes of the velocity potential above and below the surface, and $\tilde{V}\left(P\right)$ is the amplitude of the velocity potential in any point of space. Then, it was assumed that point P approaches surface *S* (Figure 1).

Subsequently, the velocity potential first derivative (3) specified in the direction normal to surface *S* at any point P is calculated

(5) $$\frac{\partial \tilde{V}\left(P\right)}{\partial z_{P}}=\int_{S}\left({\tilde{V}}_{1}\left(Q\right)-{\tilde{V}}_{2}\left(Q\right)\right)\frac{\partial^{2}V^{*}\left(P,Q\right)}{\partial z_{P}\partial z_{Q}}dS_{Q}$$

The considered Neumann’s boundary conditions allows connection between the fluid velocity potential and the displacement of surface *S* by a differential relation

(6) $$\frac{\partial V\left(x,t\right)}{\partial z}=\frac{\partial w\left(x,t\right)}{\partial t},$$

wherein $w\left(x,t\right)=\tilde{w}\left(x\right)e^{i\omega t}$ is a displacement that is perpendicular to the plate domain.

Then, the hydrodynamic pressure acting on plate surface *S* can be determined and given by the fluid’s density coupled with the first derivative of the fluid velocity potential with respect to time

(7) $$p=-\rho_{f}\frac{\partial V\left(x,t\right)}{\partial t}.$$

Now, Equation (5) can be differentiated again with respect to time, and the above expressions (6) and (7) are taking place for it. The above operations lead to the relation between the circular frequencies of a fluid and amplitude of displacement with the amplitude of the resultant hydrodynamic pressure at point Q placed on surface *S*

(8) $$-\rho_{f}\omega^{2}\tilde{w}\left(P\right)=\int_{S}\Delta \tilde{p}\left(Q\right)\frac{\partial^{2}V^{*}\left(P,Q\right)}{\partial z_{P}\partial z_{Q}}dS_{Q},$$

wherein $\Delta \tilde{p}\left(Q\right)={\tilde{p}}_{2}\left(Q\right)-{\tilde{p}}_{1}\left(Q\right)$ is the difference in hydrodynamic pressure between the upper and lower surfaces of the plate.

**Figure 2:**
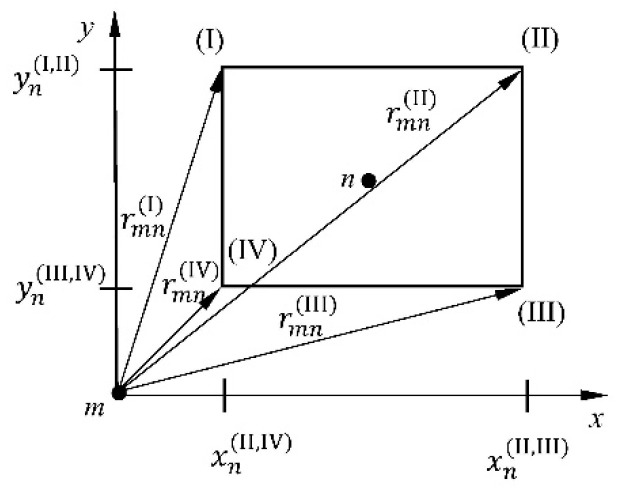
Designations of rectangular subsurface for the fluid.

The boundary integral in Equation (8) can be solved using the Boundary Element Method (BEM). The fluid surface adjacent to the plate is divided into a finite number of subsurfaces that act as boundary elements for the fluid. Using the simplest approximation for a rectangular plate, each subsurface has only one collocation point, which is placed centrally (Figure 2).

The plate domain is divided into a finite number of subdomain surfaces with areas *S_n_*, and the amplitude of displacement in arbitrary points (*x_m_*, *y_m_*) are coupled with the hydrodynamic pressure amplitude

(9) $$\omega^{2}\tilde{w}\left(x_{m},y_{m}\right)=-\frac{1}{4\pi \rho_{f}}\cdot \sum_{n=1}^{N}\Delta {\tilde{p}}_{n}\left(\int_{S_{n}}\frac{\partial^{2}}{\partial z_{m}^{2}}\left[\frac{1}{r}\right]\right)\cdot \underset{z\to 0}{dS_{n}}.$$

The boundary integral Equation (9) can be rewritten in the matrix notation to the form

(10) $$-4\pi \rho_{f}\omega^{2}\tilde{w}=H\tilde{p}$$

and **H** is the (*N* × *N*) matrix with elements derived as the integral over the *n*th subsurface, wherein *N* expresses the total number of internal subsurfaces coupled with single collocation points (subsurfaces of the constant type) [22,23].

(11) $$H_{mn}=\int_{S_{n}}\frac{\partial^{2}}{\partial z_{m}^{2}}\left[\frac{1}{r}\right]\cdot \underset{z\to 0}{dS_{n}}.$$

Now, the hydrodynamic forces acting on the plate surface can be represented as:

(12) $$P=-M_{f}\omega^{2}w$$

where **P** is the vector of the resultant hydrodynamic forces, where each element is defined as $P_{n}=\Delta p_{n}S_{n}$, and **M**_f_ is the fluid mass matrix. This matrix is expressed by the relation

(13) $$M_{f}=4\pi \rho_{f}SH^{-1}$$

with the diagonal matrix **S** which groups areas of internal subdomains

(14) $$S=\mathrm{diag}\left(S_{1}...S_{N}\right)$$

All of the elements of the matrix **H** can be calculated analytically, e.g., [22,46], according to designations presented in Figure 2

(15) $$H_{mn}=-\frac{1}{x_{n}^{\left(III,IV\right)}}\left(\frac{x_{n}^{\left(II,III\right)}}{r_{mn}^{\left(II\right)}}-\frac{x_{n}^{\left(II,IV\right)}}{r_{mn}^{\left(I\right)}}\right)+\frac{1}{x_{n}^{\left(II,III\right)}}\left(\frac{y_{n}^{\left(I,II\right)}}{r_{mn}^{\left(III\right)}}-\frac{{y_{n}^{\left(III,IV\right)}}_{p}}{r_{mn}^{\left(II\right)}}\right)+\;+\frac{1}{y_{n}^{\left(I,II\right)}}\left(\frac{x_{n}^{\left(II,III\right)}}{r_{mn}^{\left(III\right)}}-\frac{x_{n}^{\left(II,IV\right)}}{r_{mn}^{\left(IV\right)}}\right)-\frac{1}{x_{n}^{\left(II,IV\right)}}\left(\frac{y_{q}}{r_{mn}^{\left(IV\right)}}-\frac{y_{p}}{r_{mn}^{\left(I\right)}}\right)$$

Next, a description of the problem of two plates surrounded by liquid is presented. The problem of the dynamics of a single plate interacting with a liquid can be extended to the problem of the dynamics of a finite number of plates immersed in a liquid medium.

**Figure 3:**
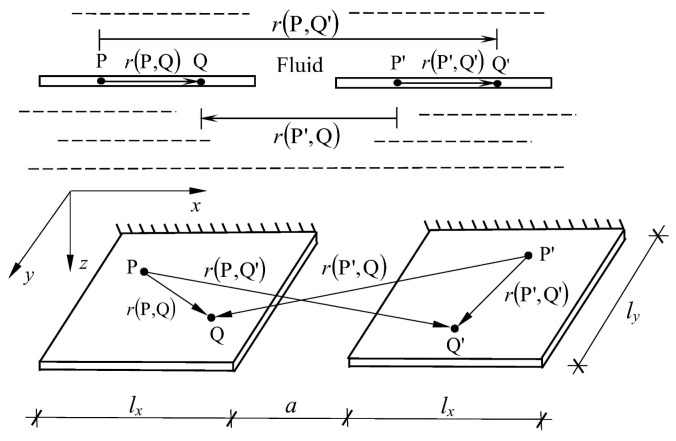
A system of two plates placed in one plane and immersed in fluid.

**Figure 4:**
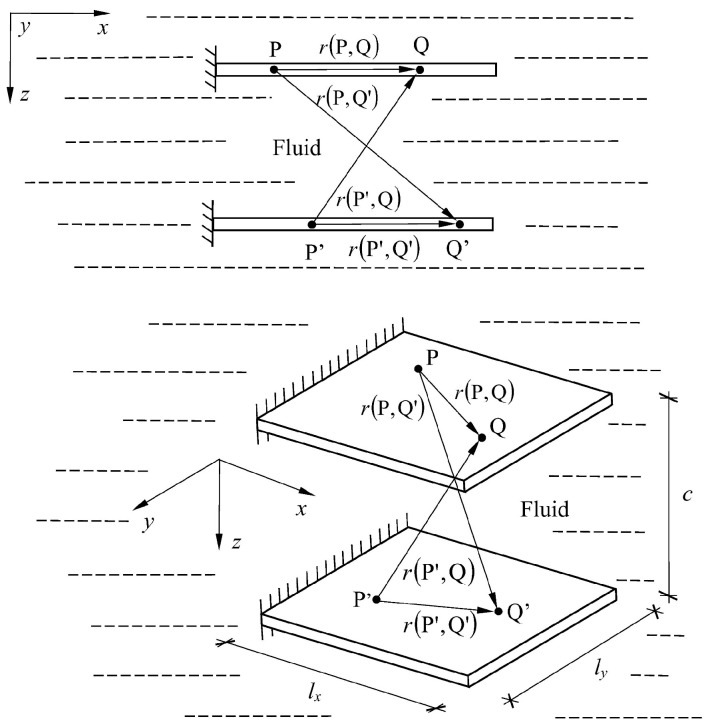
A system of two plates placed one above another and immersed in fluid.

A medium constraint in the form of a rigid partition, tank bottom, or free surface may also be assigned to such a system. This issue is illustrated using an example of natural and forced vibrations of a system of two plates immersed in a liquid medium. A system of two plates immersed in a liquid medium is presented. The plates were situated in the same plane (Figure 3) or one above another (Figure 4).

Therefore, it is necessary to construct the matrix **H** in the modified form:

(16) $$H{}^{\left(1,2\right)}=\left[\begin{array}{cc} H\left(P,Q\right) & H\left(P,Q^{\prime }\right)\\ H\left(P^{\prime },Q\right) & H\left(P^{\prime },Q^{\prime }\right) \end{array}\right]$$

where matrices $H\left(P,Q\right)$, $H\left(P,Q^{\prime }\right)$, $H\left(P^{\prime },Q\right)$, and $H\left(P^{\prime },Q^{\prime }\right)$ consist of surface integrals from the fundamental solution (12) over the subsurfaces belonging to the first or the second plate.

In view of the above, the fluid mass matrix for the system of two plates interacting with a liquid is defined:

(17) $$M_{f}^{\left(1,2\right)}=4\pi \rho_{f}S^{\left(1,2\right)}{\left[H^{\left(1,2\right)}\right]}^{-1}$$

where $S^{\left(1,2\right)}$ groups the areas of the interior subsurfaces of the first and second plates, respectively.

## 3. Applied Numerical Solutions

### 3.1. The Boundary Element Method Solution

A thin (Kirchhoff–Love) plate is surrounded by a fluid, and its vibrations have small amplitudes. Inside the plate domain, additional collocation points, coupled with lumped masses, were introduced according to Bèzine’s approach [9]. In each collocation occurring inside a plate domain, vectors of displacements $w_{i}\left(t\right)$, accelerations ${\ddot{w}}_{i}\left(t\right)$, and inertial forces $B_{i}\left(t\right)$ dependent on time *t* were introduced, e.g., [22,23]

(18) $$w_{i}\left(t\right)={\tilde{W}}_{i}\sin\omega t,\;{\ddot{w}}_{i}\left(t\right)=-\omega^{2}{\tilde{W}}_{i}\sin\omega t,\;B_{i}\left(t\right)={\tilde{B}}_{i}\sin\omega t,$$

where the amplitude of a plate’s inertial force is given by ${\tilde{B}}_{i}=\omega^{2}m_{i}W_{i}$, $m_{i}$ is a lumped mass, $W_{i}$ is the amplitude of displacement, and ω is the natural frequency of the plate vibrations.

The boundary and domain integral equations describing the plate deformation have the characteristics of amplitude equations in terms of the modified approach of a thin plate bending description, e.g., [45]. These equations may be derived using, e.g., Betti’s theorem, or otherwise they are integral representations of the thin plate differential equation solution

(19) $$\begin{array}{c} c\left(x\right)\tilde{w}\left(x\right)+\int_{\Gamma }\left[T_{n}^{*}\left(y,x\right)\tilde{w}\left(y\right)-M_{ns}^{*}\left(y,x\right)\frac{d\tilde{w}\left(y\right)}{ds}-M_{n}^{*}\left(y,x\right){\tilde{\phi }}_{n}\left(y\right)\right]d\Gamma \left(y\right)=\\ =\int_{\Gamma }\left[{\tilde{T}}_{n}\left(y\right)w^{*}\left(y,x\right)-{\tilde{M}}_{n}\left(y\right)\phi_{n}^{*}\left(y,x\right)\right]d\Gamma \left(y\right)+\sum_{j=1}^{N}{\tilde{P}}_{j}w^{*}\left(y,x\right), \end{array}$$

(20) $$\begin{array}{c} c\left(x\right){\tilde{\phi }}_{n}\left(x\right)+\int_{\Gamma }\left[{\overset{-}{T}}_{n}^{*}\left(y,x\right)\tilde{w}\left(y\right)-{\overset{-}{M}}_{ns}^{*}\left(y,x\right)\frac{dw\left(y\right)}{ds}-{\overset{-}{M}}_{n}^{*}\left(y,x\right){\tilde{\phi }}_{n}\left(y\right)\right]d\Gamma \left(y\right)=\\ =\int_{\Gamma }\left[{\tilde{T}}_{n}\left(y\right){\overset{-}{w}}^{*}\left(y,x\right)-{\tilde{M}}_{n}\left(y\right){\overset{-}{\phi }}_{n}^{*}\left(y,x\right)\right]d\Gamma \left(y\right)+\sum_{j=1}^{N}{\tilde{P}}_{j}{\overset{-}{w}}^{*}\left(y,x\right), \end{array}$$

where $w^{*}\left(y,x\right)=\left(1/\left(8\pi D\right)\right)r^{2}\ln\left(r\right)$ is the Green function, which is the fundamental solution of the biharmonic equation $\nabla^{4}w^{*}\left(y,x\right)=\left(1/D\right)\delta \left(y,x\right)$ for a thin isotropic plate, δ is the Dirac delta, $r=\left|y-x\right|$, **x** is the source point, **y** is a field point, $D=Eh^{3}/\left[12\left(1-v^{2}\right)\right]$ is the plate stiffness, *h* is the plate thickness, and *E* and *v* are Young’s modulus and Poisson’s ratio, respectively. The relation between the angle of rotation $\phi_{s}\left(y\right)$ and deflection $w\left(y\right)$ is specified by the differential relationship $\phi_{s}\left(y\right)=dw\left(y\right)/ds$, e.g., [22]. The Kirchhoff corner forces were expressed (replaced) by the forces distributed along the boundary elements placed in the vicinity of the plate corners. Variables $T_{n}^{*}\left(y,x\right)$, $M_{n}^{*}\left(y,x\right)$, $M_{ns}^{*}\left(y,x\right)$, ${\overset{-}{T}}_{n}^{*}\left(y,x\right)$, ${\overset{-}{M}}_{n}^{*}\left(y,x\right)$, and ${\overset{-}{M}}_{ns}^{*}\left(y,x\right)$ denote the fundamental functions were derived directly from the fundamental solution $w^{*}\left(y,x\right)$ as its derivatives, according to the thin plate (Kirchhoff–Love) theory. The *j*th amplitude of the resultant inertial forces ${\tilde{P}}_{j}={\tilde{B}}_{j}+{\tilde{B}}_{j}^{f}$ occurring in Equations (19) and (20) is responsible for the coupled inertial forces of a plate–fluid system, e.g., [22,23,45]. The value of the coefficient $c\left(x\right)$ occurring in Equations (20) and (21) is 1 for **x** located inside the plate domain, 0.5 for **x** located on the smooth boundary, and 0 for **x** located outside the plate domain.

**Figure 5:**
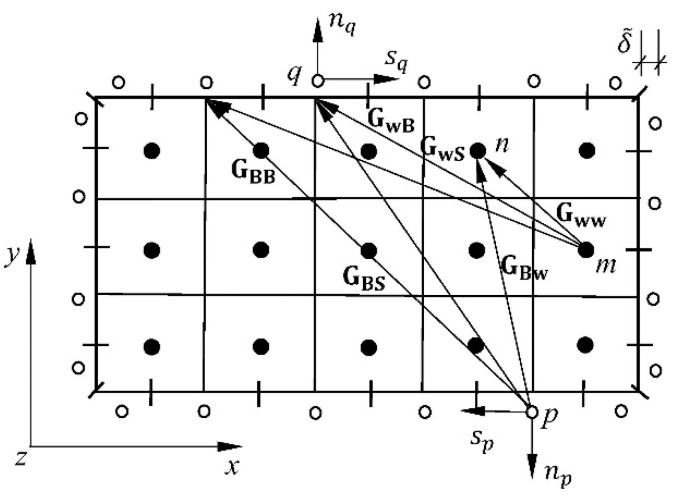
Designations used in the assembly of the set of algebraic equations.

The BEM methodology was applied with the discretization of a plate edge using boundary elements, in this case, of the constant type, and a plate domain was divided into rectangular subsurfaces coupled with one collocation point as shown in Figure 5. Each of these plate subsurfaces was coupled with the corresponding ones describing the acting liquid.

In view of the above, the system of equations has the form

(21) $$\left[\begin{array}{cc} {\overset{-}{G}}_{BB}^{\left(1,2\right)} & -\lambda \cdot G_{Bw}^{\left(1,2\right)}\cdot M^{\left(1,2\right)}\\ {\overset{-}{G}}_{wB}^{\left(1,2\right)} & -\lambda \cdot G_{ww}^{\left(1,2\right)}\cdot M^{\left(1,2\right)}+I \end{array}\right]\cdot \left\{\begin{array}{c} \tilde{X}{}^{\left(1,2\right)}\\ \tilde{w}{}^{\left(1,2\right)} \end{array}\right\}=\left\{\begin{array}{c} 0\\ 0 \end{array}\right\}$$

and the vector

(22) $${\tilde{X}}^{\left(1,2\right)}={\left\{\begin{array}{cc} {\tilde{X}}^{\left(1\right)} & {\tilde{X}}^{\left(2\right)} \end{array}\right\}}^{T}$$

groups the amplitudes of the boundary quantities:

(23) $$X^{\left(1\right)}={\left\{\begin{array}{cc} {\tilde{B}}^{\left(1\right)} & {\tilde{\phi }}_{S}^{\left(2\right)} \end{array}\right\}}^{T}X^{\left(2\right)}={\left\{\begin{array}{cc} {\tilde{B}}^{\left(2\right)} & {\tilde{\phi }}_{S}^{\left(2\right)} \end{array}\right\}}^{T}.$$

The vector

(24) $${\tilde{w}}^{\left(1,2\right)}={\left\{\begin{array}{cc} {\tilde{w}}^{\left(1\right)} & {\tilde{w}}^{\left(2\right)} \end{array}\right\}}^{T}$$

is formed by the amplitudes of the internal displacements of the collocation points and

(25) $$G_{BB}^{\left(1,2\right)}=\left[\begin{array}{cc} {\overset{-}{G}}_{BB}^{\left(1\right)} & 0\\ 0 & {\overset{-}{G}}_{BB}^{\left(2\right)} \end{array}\right],\;G_{Bw}^{\left(1,2\right)}=\left[\begin{array}{cc} G_{Bw}^{\left(1\right)} & 0\\ 0 & G_{Bw}^{\left(2\right)} \end{array}\right],\;G_{wB}^{\left(1,2\right)}=\left[\begin{array}{cc} {\overset{-}{G}}_{wB}^{\left(1\right)} & 0\\ 0 & {\overset{-}{G}}_{wB}^{\left(2\right)} \end{array}\right],\;G_{ww}^{\left(1,2\right)}=\left[\begin{array}{cc} G_{ww}^{\left(1\right)} & 0\\ 0 & G_{ww}^{\left(2\right)} \end{array}\right],$$

where matrices ${\overset{-}{G}}_{BB}^{\left(j\right)}$ and ${\overset{-}{G}}_{wB}^{\left(j\right)}$ contain appropriate boundary integrals for a single plate (*j* = 1, 2) depending on the type of boundary. In the matrix $G_{Bw}^{\left(j\right)}$, the values of the basic functions $w^{*}\left(y,x\right)$ (this function comes from the unit concentrated force) and ${\overset{-}{w}}^{*}\left(y,x\right)$ (this function is taken from the unit moment normal to the plate boundary) calculated in the coordinate system related to the current physical node (boundary element) of the *j*^th^ plate are grouped. Matrix $G_{ww}^{\left(j\right)}$ consists of the values of the basic function calculated in the coordinate system related to the current internal collocation point. The matrix is expressed as the sum of

(26) $$M^{\left(1,2\right)}=M_{p}^{\left(1,2\right)}+M_{f}^{\left(1,2\right)}$$

where

(27) $$M_{p}^{\left(1,2\right)}=\left[\begin{array}{cc} M_{p}^{\left(1\right)} & 0\\ 0 & M_{p}^{\left(2\right)} \end{array}\right]$$

and $M_{p}^{\left(j\right)}=\mathrm{diag}\left(m_{1}^{\left(j\right)},m_{2}^{\left(j\right)},...,m_{N}^{\left(j\right)}\right)$, $\lambda =\omega^{2}$, **I** is the unit matrix, and N is the number of lumped masses for a single plate. Elimination of the boundary variables ${\tilde{X}}^{\left(1,2\right)}$ from the matrix Equation (22) leads to the standard eigenvalue problem

(28) $$\left\{A^{\left(1,2\right)}-\tilde{\lambda }\cdot I\right\}w^{\left(1,2\right)}=0,$$

where $\tilde{\lambda }=1/\omega^{2}$ and

(29) $$A^{\left(1,2\right)}=\left\{G_{ww}^{\left(1,2\right)}\cdot M^{\left(1,2\right)}-G_{wB}^{\left(1,2\right)}\cdot {\left[G_{BB}^{\left(1,2\right)}\right]}^{-1}\cdot G_{Bw}^{\left(1,2\right)}\cdot M^{\left(1,2\right)}\right\}$$

Note the fundamental function used to compute the matrix $H^{\left(1,2\right)}$ elements. This function is obtained by performing a double differentiation of the function 1/r with respect to the *z* coordinate, where $r=\sqrt{x^{2}+y^{2}+z^{2}}$ [22,45].

### 3.2. The Finite Element Method Approach to the Fluid–Structure Interaction

The free vibration problem of the structure can be described by a generalized eigenvalue problem, which can be written in matrix notation as follows

(30) $$\left(K^{\left(1,2\right)}-\omega^{2}M^{\left(1,2\right)}\right){\tilde{w}}^{\left(1,2\right)}=0,$$

where $K^{\left(1,2\right)}$ and $M^{\left(1,2\right)}$ are the stiffness and mass matrices of the whole system, respectively, *ω* is the eigenvalue, and ${\tilde{w}}^{\left(1,2\right)}$ is the nonzero eigenvector. The matrices $K^{\left(1,2\right)}$ and $M^{\left(1,2\right)}$ have the form

(31) $$K^{\left(1,2\right)}=\left[\begin{array}{cc} K^{\left(1\right)} & 0\\ 0 & K^{\left(2\right)} \end{array}\right],\;M^{\left(1,2\right)}=\left[\begin{array}{cc} M_{11} & M_{12}\\ M_{21} & M_{22} \end{array}\right],$$

where $K^{\left(1\right)}$ and $K^{\left(2\right)}$ are the stiffness matrices of the first and second plates, respectively, and the mass matrix of the plate–fluid system is coupled with the fluid according to relation (17). Vector ${\tilde{w}}^{\left(1,2\right)}$ has already been defined in Equation (24).

The bending of a single plate with constant thickness is described by a rectangular four-node finite element with three degrees of freedom at each node. At each element *i*^th^ node, in the Cartesian coordinate system, there are introduced: deflection *w_i_* and two angles of rotation in mutually perpendicular directions *φ_ix_* and *φ_iy_*, respectively, wherein the function of deflection is expressed as the polynomial of the fourth order [51]

(32) $$w\left(x,y\right)=\alpha_{1}+\alpha_{2}x+\alpha_{3}y+\alpha_{4}x^{2}+\alpha_{5}xy+\alpha_{6}y^{2}+\alpha_{7}x^{3}+\;+\alpha_{8}x^{2}y+\alpha_{9}xy^{2}+\alpha_{10}y^{3}+\alpha_{11}yx^{3}+\alpha_{12}xy^{3}$$

where $\alpha_{11}\neq 0$ or $\alpha_{12}\neq 0$.

The displacement field inside the finite element is presented in the FEM methodology

(33) $$w^{e}\left(x,y\right)=N_{j}^{\left(i\right)}w_{j}^{\left(i\right)}=N^{e}w^{e};i=1,2,3,4;j=1,2,3,$$

*i* expresses the number of the actual node, and *j* expresses the current degree of freedom at the *i*th node. The set of shape functions $N_{j}^{\left(i\right)}$ for each node can be given by formulas derived directly from relation (32). A detailed description of the considered finite element was presented by Kuczma [51] and quoted by Lenartowicz and Guminiak [48].

The stiffness matrix of the finite element is defined according to FEM methodology

(34) $$K_{e}=\int_{V}B^{T}DBdV=h\int_{A}B^{T}DBdA,$$

where *h* is the plate thickness, **B** is the shape functions derivatives matrix, and **D** is the plate physical quantities matrix. The mass matrix of the single element **M**_e_ has the characteristics of the consistent matrix. The boundary conditions for the plates were introduced according to the well-known FEM methodology.

### 3.3. The Finite Difference Method Solution to the Fluid–Structure Interaction

The free vibration in terms of the Finite Difference Method is described by the difference procedures leading to the following matrix equation which has the characteristics of the generalized eigenvalue problem

(35) $$\left({K_{\Delta }}^{\left(1,2\right)}-\omega^{2}M^{\left(1,2\right)}\right){\tilde{w}}^{\left(1,2\right)}=0.$$

**Figure 6:**
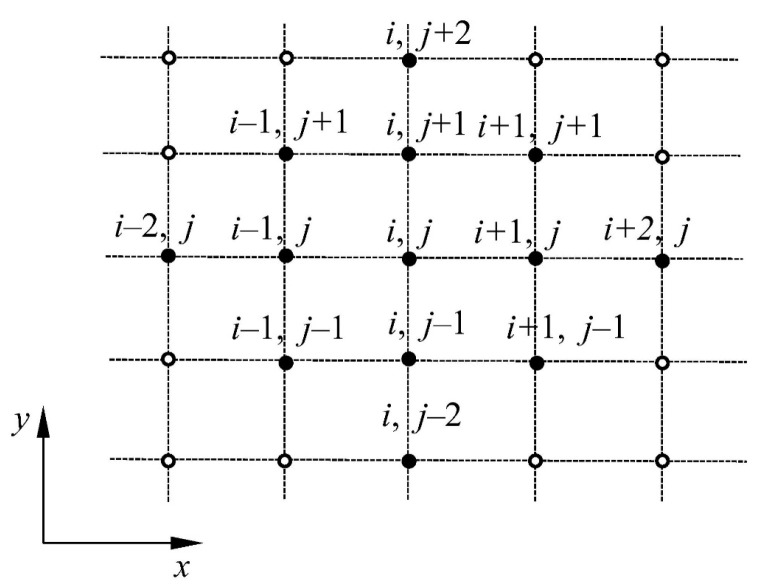
The set of the finite difference points dividing the single plate area.

The matrix of suitable difference operators for the system of two plates $K_{\Delta }^{\left(1,2\right)}$ has the form

(36) $$K_{\Delta }^{\left(1,2\right)}=\left[\begin{array}{cc} K_{\Delta }^{\left(1\right)} & 0\\ 0 & K_{\Delta }^{\left(2\right)} \end{array}\right]$$

and group matrices of the difference operators of the first and second plates $K_{\Delta }^{\left(1\right)}$ and $K_{\Delta }^{\left(2\right)}$ (Figure 6) The mass matrix of the plate–fluid system was coupled with the fluid according to relation (17); the mass matrix of a single plate **M** is a diagonal matrix containing plate masses m_i_, which are concentrated in subsequent FDM mesh nodes, divided by their surface area. Finally, the vector ${\tilde{w}}^{\left(1,2\right)}$ is expressed by the relation (24).

The boundary conditions for the plates are introduced according to the FDM methodology, described comprehensively [48].

## 4. Probabilistic Responses and Relative Entropy Application

The Gaussian uncertainty in some of the materials or geometrical design parameters of the aforementioned plate was assumed. Random quantities include the thickness of the plate, Young’s modulus, Poisson’s ratio, distance of the collocation point from the plate edge, boundary conditions, and others. The structural response functions were realized using polynomial curves of the third order. Structural uncertainty numerical analysis of the considered plates was performed using the semi-analytical method (SAM), iterative generalized 10th order stochastic perturbation technique (SPT), and Monte Carlo simulation (MCS).

First, for the SAM approach, the probability distribution of the observed quantity must be assumed in advance. This can be, for example, a Gaussian distribution. Next, the polynomial expressions should be applied directly to the probabilistic integral formulas, which allows the analytical calculation of all of the formulas for the expected values, coefficients of variation, etc., to have been implemented due to the derivations presented in [49]. This approach is relatively simple and easy to implement.

A more complex approach is the Stochastic Perturbation Technique (SPT). In this study, the Taylor series expansion of all the input variables and state functions was used, assuming that the perturbation parameter is ε. This expansion is then used to determine random moments. Random moments can be calculated analytically or determined in symbolic form if and only if the function of the random parameter is known.

The Monte Carlo simulation approach appears to be the simplest because it is based on the so-called almost large numbers; unfortunately, it entails a large number of operations (trials) on the order of fifty or one hundred thousand. However, in the case of a classical numerical simulation, a certain probability distribution of the occurrence of a random quantity should be assumed. Therefore, this method is characterized by a high time cost.

*R* denotes the admissible limit of the given structure, and *E* denotes its extreme effort. The previous engineering design codes allowed us to make the following interpretations in the case of the eigenfrequency and extreme excitation. The distance in between them cannot be smaller than a quarter of this eigenfrequency. This was carried out to avoid structural resonance. Therefore, the satisfactory safety of the given system may be measured using the following FORM reliability index:

(37) $$\beta_{\mathrm{FORM}}=\frac{E\left[R\right]-E\left[E\right]}{\sqrt{Var\left(R-E\right)}}=\frac{E\left[\omega_{i}\right]-E\left[\frac{3}{4}\omega_{i}\right]}{\sigma \left(\omega_{i}-\frac{3}{4}\omega_{i}\right)},$$

where $\omega_{i}$ stands for each next eigenfrequency. Quite a similar calculation can be provided with the use of a relative entropy *H*. This entropy can be calculated due to the Bhattacharyya theory as

(38) $$H=\frac{1}{4}\frac{{\left(E\left[R\right]-E\left[E\right]\right)}^{2}}{\sigma^{2}\left(R\right)+\sigma^{2}\left(E\right)}+\frac{1}{2}\ln\left(\frac{\sigma^{2}\left(R\right)+\sigma^{2}\left(E\right)}{2\sigma \left(R\right)\sigma \left(E\right)}\right).$$

This can be rewritten as

(39) $$H\left(\omega_{i}\right)=\frac{1}{4}\frac{{\left(E\left[\omega_{i}\right]-E\left[\frac{3}{4}\omega_{i}\right]\right)}^{2}}{\sigma^{2}\left(\omega_{i}\right)+\sigma^{2}\left(\frac{3}{4}\omega_{i}\right)}+\frac{1}{2}\ln\left(\frac{\sigma^{2}\left(\omega_{i}\right)+\sigma^{2}\left(\frac{3}{4}\omega_{i}\right)}{2\sigma \left(\omega_{i}\right)\sigma \left(\frac{3}{4}\omega_{i}\right)}\right).$$

The final safety measure compensable to *β* form is obtained as

(40) $$\beta_{H}=\frac{1}{2}\sqrt{H\left(\omega_{i}\right)}.$$

## 5. Numerical Examples

The natural vibrations of the system of two thin elastic plates surrounded by liquid on all sides were considered. The system of plates was analyzed numerically using three independent Stochastic Boundary Element Methods (SBEM). Numerical comparison of the deterministic BEM, FEM, and FDM solutions for the first four fundamental natural frequencies for a plate immersed in fluid were performed as the foundations for further SBEM, SFEM, and SFDM procedures with Young’s modulus equal to *E* = 205 GPa, Poisson’s ratio *v* = 0.3, plate dimensions *l_x_* = *l_y_* = 2.0 m, plate thickness h = 0.05 m, and fluid density *ρ*_f_ = 1000 kg/m^3^. The numerical results were compared with the analytical solutions obtained by C.C. Liang et al. [44] and Lenartowicz and Guminiak [48] for a single plate fully submerged in fluid.

The BEM-BEM numerical computations were carried out using the original computational program developed by the first author of the Fortran 90 programming language. The direct version of the BEM and the static fundamental solution of the Kirchhoff plate problem were used, where the quasi-diagonal boundary terms in the characteristic matrix were calculated analytically, and the nondiagonal terms were calculated numerically by applying a 12-point Gauss quadrature. The angle of rotation in the tangent direction occurring on the free edge was calculated by formulating the difference relations between displacements for three boundary nodes (collocation points) taking place next to each other.

The FEM-BEM and FDM-BEM deterministic computations were processed using the numerical procedures developed by the last two authors of the OCTAVE numerical package. Finally, probabilistic analyses were performed using the original procedures developed by the second author of the MAPLE v.19 scientific package, where LSM fittings and three probabilistic approaches (SAM, SPT, and MCS) were implemented. The following basic discretization of a single plate was taken: the number of boundary elements was 100, the number of internal subdomains was 100, the number of finite elements was 256, and the number of finite difference grids was 900 for the BEM-BEM, FEM-BEM, and FDM-BEM approaches, respectively.

For probabilistic investigation, the following intervals of variables and their representations for the needs of the LSM analysis are presented: $\tilde{\delta }$ varies from 0.0001 m to 0.025 m forming the series [0.0001, 0.00015, 0.0002, 0.00025, 0.0005, 0.001, 0.005, 0.01, 0.015, 0.02, 0.025], the fluid density takes values from 900 to 1100 kg/m^3^ and its increase is 20 kg/m^3^, the plate thickness changes from 0.04 m to 0.06 m with an increment of 0.002 m, Young’s modulus takes values from 185 to 235 kN/m^2^ with an increase of 5 kN/m^2^, and Poisson’s ratio increases from 0.25 to 0.35 with increments equal to 0.01.

The impact of the parameter $\tilde{\delta }$ on the structural response for the first four natural frequencies of the single cantilever plate fully submerged in fluid for the BEM-BEM solution and comparison with the analytical FEM-BEM and FDM-BEM solutions are presented in Table 1. It is clear from these results that the BEM-BEM approach generally returns all the basic natural frequencies larger than the analytical method, yet still smaller than the combined FEM-BEM and FDM-BEM approaches. Moreover, an influence of the parameter $\tilde{\delta }$ is rather marginal because even dramatic changes of its mean value result in negligible modifications of the aforementioned frequencies. This is an important result, which shows no sensitivity from the BEM approach towards the numerical parameter of the plate(s) discretization.

### 5.1. Two Cantilever Plates Immersed in a Fluid and Placed in One Plane along One Line

**Figure 7:**
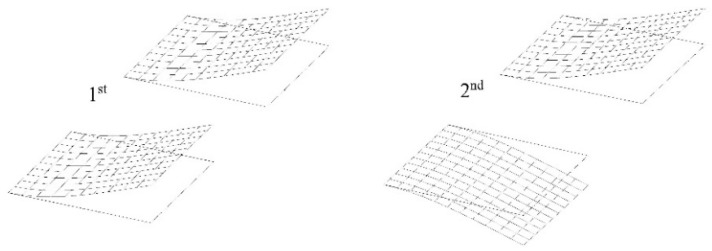
Two modes of two cantilever plates immersed in a fluid and placed in one plane along one line for the BEM-BEM solution.

First, the results of two modes of natural vibrations of the system of cantilever plates fully submerged in water from the BEM-BEM solution and *a* = 1.0 m are shown in Figure 7. The influence of parameter $\tilde{\delta }$ on the structural response for the first four natural frequencies of the two cantilever plates fully submerged in fluid for the BEM-BEM solution is presented in Table 2. A comparison of the results from the FEM-BEM and FDM-BEM solutions is presented in Table 3 for basic discretization using a 16×16 plate FEM mesh with a 15×15 fluid BEM mesh and a 30×30 plate FDM grid with a 15×15 fluid BEM mesh.

Table 4 and Table 5 show comparisons of the results obtained from the three different plate FEM meshes, plate FDM grids, and fluid BEM meshes.

The results presented in these tables lead to almost the same conclusion as the data included in Table 1; not a single plate, nor the system of two plates in fluid show any sensitivity to the parameter $\tilde{\delta }$. The BEM-BEM solution returns the smallest values of the given eigenfrequency each time slightly larger values are noticed in the FEM-BEM coupling, and the largest results are observed in the FDM-BEM solution. This means that the results of the BEM-BEM approach can be used for reliability assessment in the worst scenario.

#### 5.1.1. Stochastic BEM-BEM Analysis

The main aim of the first numerical experiment was to compare the proposed triple probabilistic methods: the semi-analytical method (SAM), perturbative technique (SPT), and Monte Carlo simulations (MCS). The set of deterministic problems related to the system of two plates immersed in fluid was solved using the BEM to describe the plate bending and to determine the inertial forces coming from the fluid (BEM-BEM analysis). A comparison was performed for the random placement of the distance between these plates expressed by parameter *a* for a plate exhibiting a Gaussian uncertainty natural frequency defined by its mean value and the specific range of its coefficient of variation, i.e., $\alpha \left(b\right)\in \left[0.00,0.025\right]$. The response function for the first natural frequency is expressed as the third-order polynomial:

(41) $$\omega_{1}\left(a\right)=36.7668464621162+1.24098264874659\cdot a-\;-0.815495483729244\cdot a^{2}+0.202623591700883\cdot a^{3}$$

The response function for the second natural frequency is expressed as the third-order polynomial:

(42) $$\omega_{2}\left(a\right)=38.2490136092903-0.983703196940652\cdot a+\;+0.594424679460365\cdot a^{2}-0.136650641017455\cdot a^{3}$$

**Figure 8:**
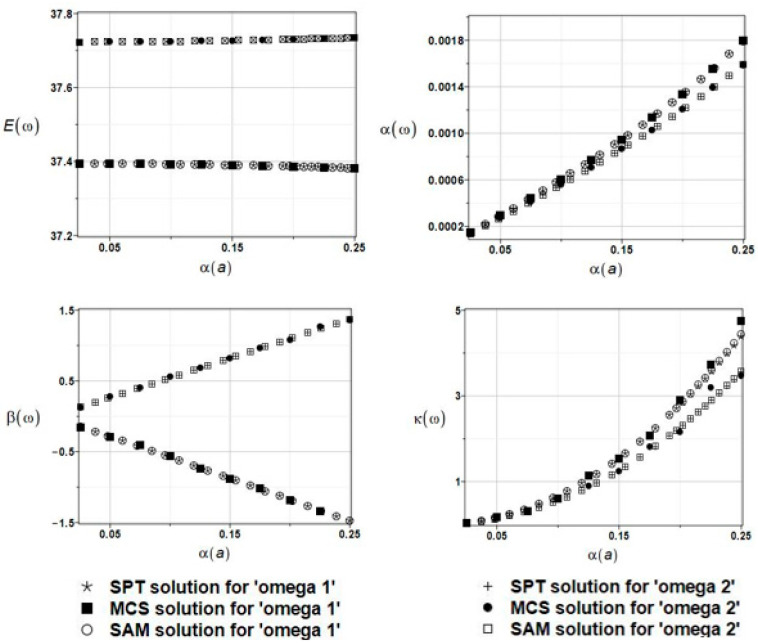
The results of the first and second natural frequencies, ω_1_ and ω_2,_ with randomly distributed placement parameter a, BEM-BEM analysis.

The results of the calculation of the expected values $E\left(\omega \right)$, coefficients of variation $\alpha \left(\omega \right)$, skewness $\beta \left(\omega \right)$, and kurtosis $\kappa \left(\omega \right)$ of the first and the second natural frequencies are presented in Figure 8.

First of all, it was observed that the three probabilistic methods return similar results each time; therefore, they can be used alternatively as a solution to this problem. Further, the influence of the uncertainty within this parameter has a negligibly small impact on the statistical scattering of the natural frequencies of the given system; the resulting coefficients of variation equal all almost 0. Finally, the resulting eigenfrequency probability distribution is rather distant from the Gaussian one because higher-order statistics clearly diverge from 0 while increasing the coefficient *CoV*(*a*).

Further, the polynomial response functions for the first two natural frequencies corresponding to varying fluid density are expressed by the third-order polynomials:

(43) $$\omega_{1}\left(\rho_{f}\right)=60.7480146145228-0.0376800457997232\cdot \rho_{f}+\;+0.000018162107183953\cdot \rho_{f}-3.83515014214677\cdot 10^{-9}\cdot {\rho_{f}}^{3}$$

(44) $$\omega_{2}\left(\rho_{f}\right)=283.857777129669-0.722165665372473\cdot \rho_{f}+\;+0.000717926891058529\cdot {\rho_{f}}^{2}-2.41868512774842\cdot 10^{-7}\cdot {\rho_{f}}^{3}$$

**Figure 9:**
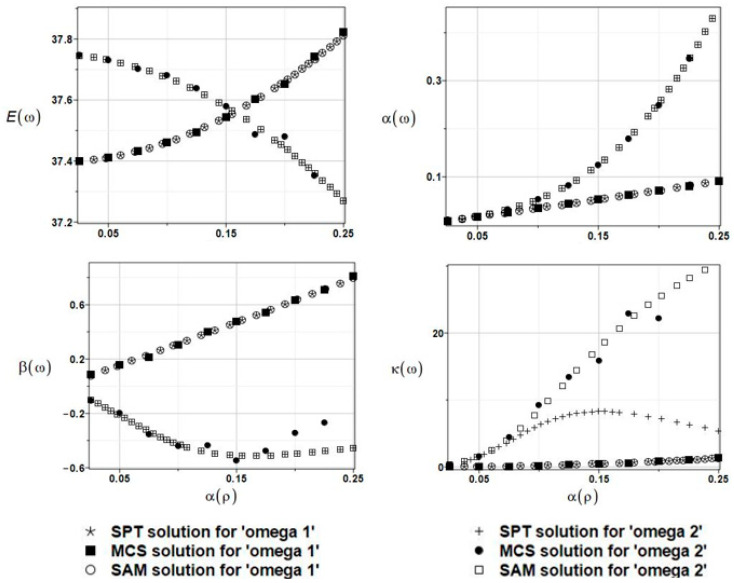
The results for the first and second natural frequencies, ω_1_ and ω_2_, with randomly distributed fluid density *ρ*_f_, BEM-BEM analysis.

The results of the basic probabilistic characteristics calculation, that is, the expected values $E\left(\omega \right)$, coefficients of variation $\alpha \left(\omega \right)$, skewness $\beta \left(\omega \right)$, and kurtosis $\kappa \left(\omega \right)$ of the first and the second natural frequencies, are presented in Figure 9 as functions of the input coefficient of variation in fluid density. This situation is remarkably different than in the case of plate thickness randomness; although the first two moments are equal when computed using different probabilistic strategies, a higher order statistics agreement is obtained for the first eigenfrequency only. Fluid density has a tremendous impact on natural frequencies, and their coefficients of variation are remarkably larger than the coefficient of variation of the fluid density.

Next, the response functions of the first two natural frequencies for the collocation point placement parameter $\tilde{\delta }$ are expressed by the third-order polynomials:

(45) $$\omega_{1}\left(\tilde{\delta }\right)=37.4048621588565-13.1254313752322\cdot \tilde{\delta }+\;+563.487878715297\cdot {\tilde{\delta }}^{2}-5259.98629104314\cdot {\tilde{\delta }}^{3}$$

(46) $$\omega_{2}\left(\tilde{\delta }\right)=37.7330701259827-13.2206635355242\cdot \tilde{\delta }+\;+567.585216001684\cdot {\tilde{\delta }}^{2}-5292.82509392625\cdot {\tilde{\delta }}^{3}$$

**Figure 10:**
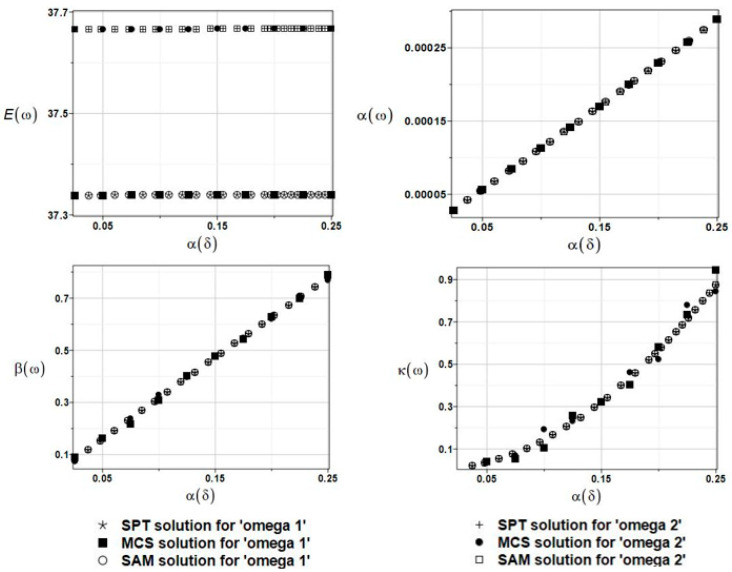
The results from the first and second natural frequencies, ω_1_ and ω_2_, with a randomly distributed collocation point placement parameter $\tilde{\delta }$, BEM-BEM analysis.

The results of the calculation with the expected values $E\left(\omega \right)$, coefficients of variation $\alpha \left(\omega \right)$, skewness $\beta \left(\omega \right)$, and kurtosis $\kappa \left(\omega \right)$ of the first and second natural frequencies are presented in Figure 10. This parameter of uncertainty had almost no statistical influence on the natural frequencies, and the PDFs of these frequencies were remarkably different from the Gaussian one.

**Figure 11:**
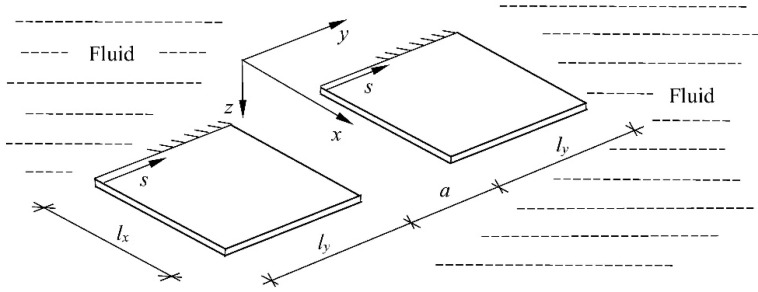
The propagation of the unsupported segment is described by the parameter “*s*”.

The next calculations were performed using a random approach on the restrained support of both plates. The propagation of the unsupported segment is described by the parameter “*s*”, as shown in Figure 11.

The response functions of the first two natural frequencies for the parameter “*s*” describing the propagation of the unsupported section of the plates are expressed by the third-order polynomials:

(47) $$\omega_{1}\left(s\right)=38.1880376080419-4.7550558937933\cdot s-\;-18.0641612398033\cdot s^{2}-10.4871553576617{\cdot s}^{3},$$

(48) $$\omega_{2}\left(s\right)=38.5197594349883-4.76747491350958\cdot s-\;-18.2226428613072\cdot s^{2}-10.5629477770474{\cdot s}^{3}.$$

**Figure 12:**
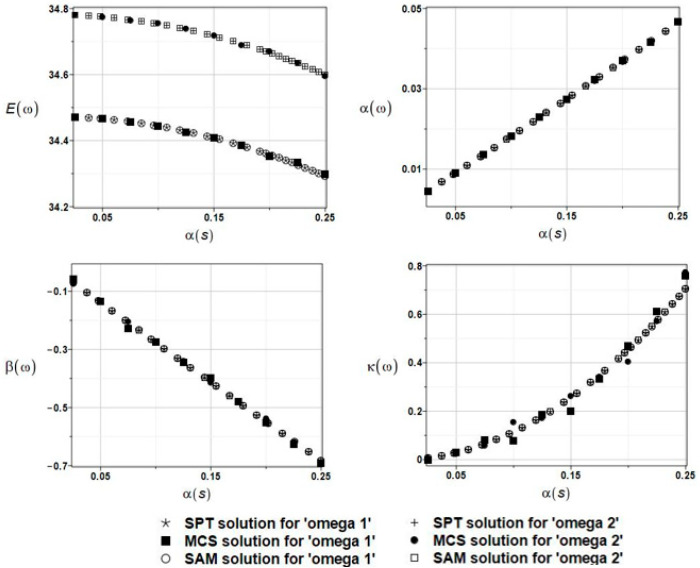
The results for the first and second natural frequencies, ω_1_ and ω_2_, with randomly distributed boundary support along the clamped edge, described by the parameter *s*, BEM-BEM analysis.

The results of the calculation of the expected values $E\left(\omega \right)$, coefficients of variation $\alpha \left(\omega \right)$, skewness $\beta \left(\omega \right)$, and kurtosis $\kappa \left(\omega \right)$ of the first and second natural frequencies are presented in Figure 12.

Now, once more SAM, SPT, and MCS probabilistic methods coincide when all characteristics are considered, whereas the uncertainty in the plate separation parameter *s* has a rather marginal influence on the random output characteristics. This impact is larger than in the case of the parameter $\tilde{\delta }$, but definitely smaller than in the case of the fluid density.

Finally, the plate thickness uncertainty was studied, where the response functions of the first two natural frequencies were approximated via the Least Squares Method by the third-order polynomials:

(49) $$\omega_{1}\left(h\right)=-3.30678625808618+565.549918102213\cdot h+\;+6117.11188665444\cdot h^{2}-22950.2037198880\cdot h^{3},$$

(50) $$\omega_{2}\left(h\right)=-3.35719158442724+573.824420091382\cdot h+\;+6105.72086246931\cdot h^{2}-23003.9797008447\cdot h^{3}.$$

**Figure 13:**
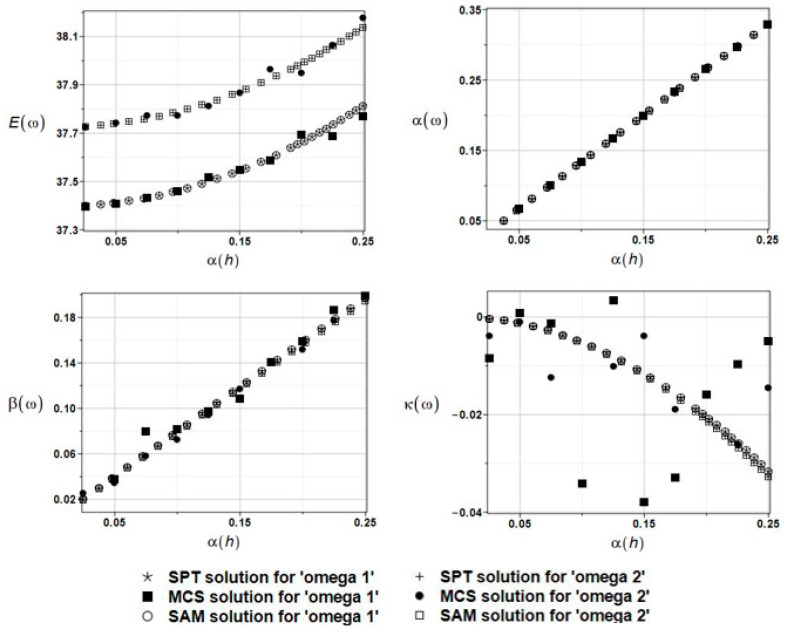
The results for the first and second natural frequencies, ω_1_ and ω_2_, with randomly distributed plate thickness *h*, BEM-BEM analysis.

The results of the calculation with the expected values $E\left(\omega \right)$, coefficients of variation $\alpha \left(\omega \right)$, skewness $\beta \left(\omega \right)$, and kurtosis $\kappa \left(\omega \right)$ of the first and second natural frequencies are presented in Figure 13. There is no doubt that this parameter should play, and really does play, a very important role in the uncertainty analysis because the output *CoV* is somewhat larger than that defined for this thickness. A coincidence of the MCS statistical estimation is discussive in case of the kurtosis only; nevertheless, the resulting eigenfrequency PDFs are close to the Gaussian density parameters.

#### 5.1.2. Stochastic FEM-BEM Analysis

In this study, the main aim of the second numerical experiment was to check probabilistic approaches by comparing the SAM, SPT, and MCS results. The deterministic problem of the system of two plates immersed in fluid was solved by using the FEM to describe plate bending and the BEM to describe the occurrence of fluid (FEM-BEM analysis). The response functions of the first two natural frequencies are expressed by the third-order polynomials:

(51) $$\omega_{1}\left(\rho_{f}\right)=61.2401331441001-0.0366305175403436\cdot \rho_{f}+\;+0.0000171736605104241\cdot {\rho_{f}}^{2}-3.5450663239711\cdot 10^{-9}\cdot {\rho_{f}}^{3}$$

(52) $$\omega_{2}\left(\rho_{f}\right)=61.3468177522571-0.0360681028239147\cdot \rho_{f}+\;+0.0000166724928758814\cdot {\rho_{f}}^{2}-3.39937796739140\cdot 10^{-9}\cdot {\rho_{f}}^{3}$$

**Figure 14:**
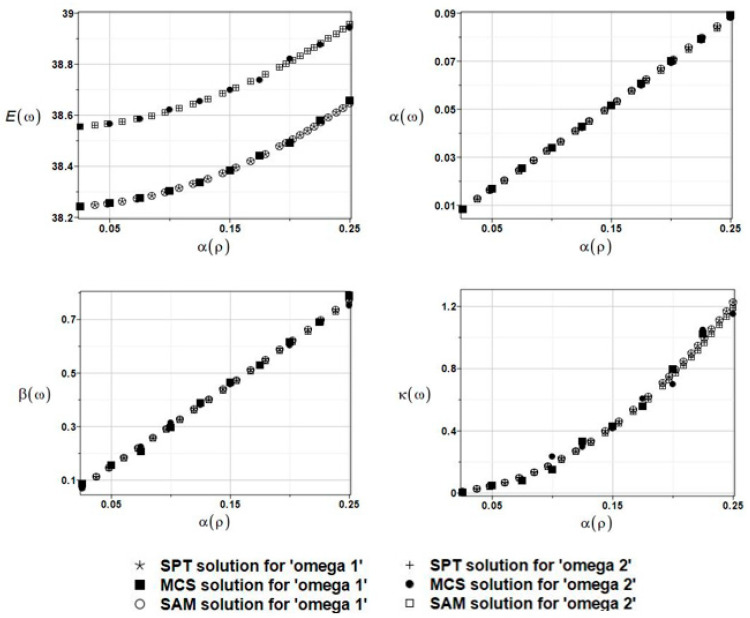
The results for the first and second natural frequencies, ω_1_ and ω_2_, with randomly distributed fluid density *ρ*_f,_ FEM-BEM analysis.

The results of the calculation with the expected values $E\left(\omega \right)$, coefficients of variation $\alpha \left(\omega \right)$, skewness $\beta \left(\omega \right)$, and kurtosis $\kappa \left(\omega \right)$ of the first and second natural frequencies are presented in Figure 14.

The three probabilistic methods coincided with each other for all probabilistic characteristics, and the induced uncertainty increased, almost linearly, while the input coefficient variation increased. The remaining characteristic of the structural response demonstrated a very similar, yet nonlinear, monotonous increase. It should be mentioned that the impact of the fluid density’s uncertainty is smaller than the one verified above, implying that the similarity of the mean values in BEM-BEM and FEM-BEM approaches does not imply any similarity in higher-order statistics. Finally, it should be pointed out that these results are extremely similar to the ones presented in Figure 14, which were obtained while replacing the FEM with the FDM.

#### 5.1.3. Stochastic FDM-BEM Analysis

Similarly, the main aim of the third numerical experiment was to validate the proposed triple probabilistic methodology by comparing the SAM, SPT, and MC approaches. The deterministic problem of the system of two plates immersed in fluid was solved using the FDM description of the plate bending and the BEM description of the occurrence fluid (FDM-BEM analysis). The response functions of the first two natural frequencies are expressed by the third-order polynomials:

(53) $$\omega_{1}\left(\rho_{f}\right)=63.2395454367537-0.0412862081827453\cdot \rho_{f}+\;+0.0000214889276855052\cdot {\rho_{f}}^{2}-4.90481738706539\cdot 10^{-9}\cdot {\rho_{f}}^{3}$$

(54) $$\omega_{2}\left(\rho_{f}\right)=61.7325032227906-0.0358022524780776\cdot \rho_{f}+\;+0.0000160314677062207\cdot {\rho_{f}}^{2}-3.10800283321458\cdot 10^{-9}\cdot {\rho_{f}}^{3}$$

**Figure 15:**
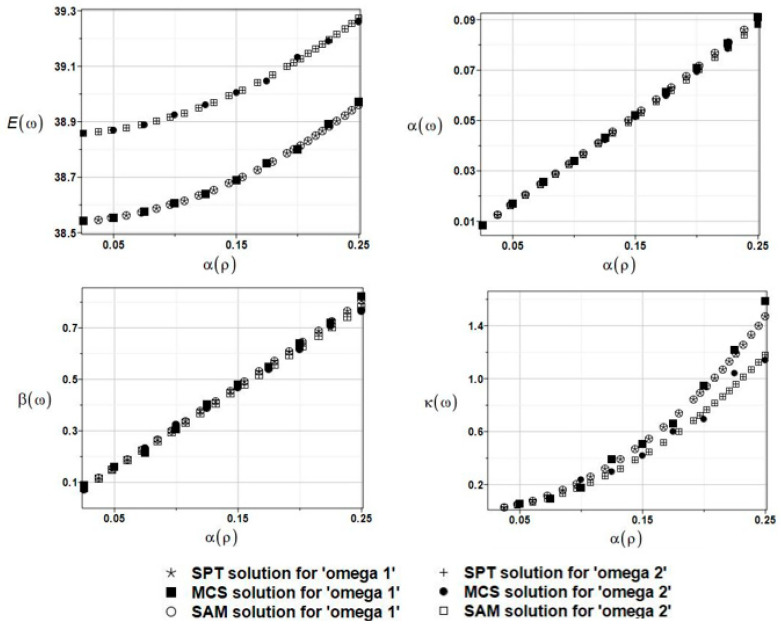
The results for the first and second natural frequencies, ω_1_ and ω_2_, with randomly distributed fluid density *ρ*_f_, FDM-BEM analysis.

The results of the calculation with the expected values $E\left(\omega \right)$, coefficients of variation $\alpha \left(\omega \right)$, skewness $\beta \left(\omega \right)$, and kurtosis $\kappa \left(\omega \right)$ of the first and the second natural frequencies are presented in Figure 15.

### 5.2. Two Cantilever Plates Immersed in Fluid and Placed One Directly above the Other

**Figure 16:**
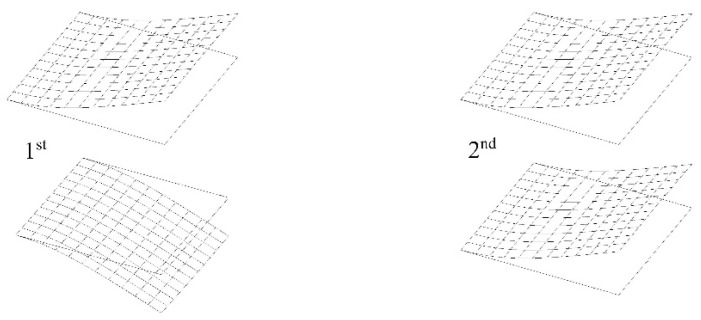
Two modes of two cantilever plates immersed in fluid and placed one above another for BEM-BEM solution.

Two modes of natural vibrations of a system of cantilever plates fully submerged in fluid (water) for the BEM-BEM solution and *c* = 1.0 m are shown in Figure 16.

The influence of the parameter $\tilde{\delta }$ on the structural response for the first four natural frequencies of the two cantilever plates surrounded by water for the BEM-BEM solution is presented in Table 6. Further, a comparison of the results for the FEM-BEM and FDM-BEM solutions and the basic discretization using a 16×16 plate FEM mesh with a 15×15 fluid BEM mesh and a 30×30 plate FDM grid with a 15×15 fluid BEM mesh are presented in Table 7. The results showed almost the same properties as in the previous section, an insensitivity to the parameter $\tilde{\delta }$, whereas an increase in the value of the natural frequencies were obtained in turn for the BEM-BEM, FEM-BEM as well as for the FDM-BEM coupled numerical strategies.

Table 8 and Table 9 show comparisons of the results obtained from three different plate FEM meshes, plate FDM grids, and fluid BEM meshes.

#### 5.2.1. Stochastic BEM-BEM Analysis

First, a comparison has been performed for the random distance placement *c* between two plates having Gaussian distribution uniquely defined by its mean value, and a specific range of the coefficient of variation, i.e., $\left[0.00,0.025\right]$. The response functions of the first two natural frequencies were obtained as the third-order polynomials:

(55) $$\omega_{1}\left(c\right)=26.9346970344194+17.5989612536558\cdot c-\;-11.5605422786156\cdot c^{2}+2.81042662200890\cdot c^{3}$$

(56) $$\omega_{2}\left(c\right)=44.0928558407688-9.10429101320153\cdot c+\;+5.21900323416288\cdot c^{2}-1.14571848287236\cdot c^{3}$$

**Figure 17:**
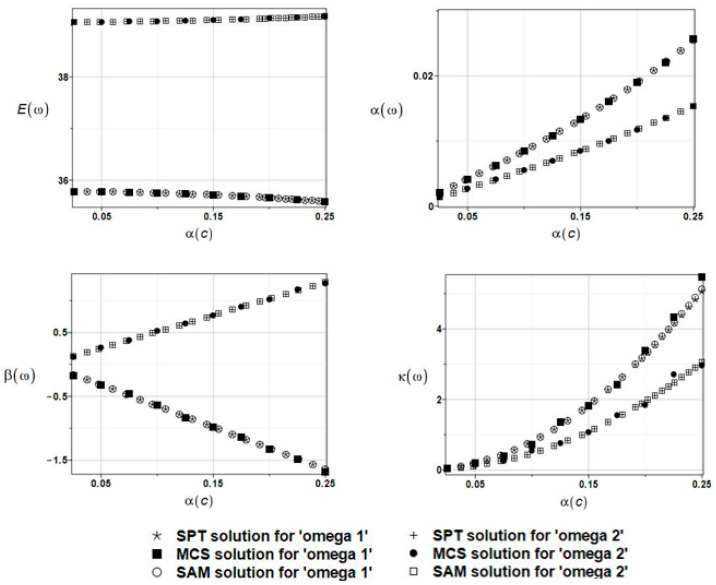
The results for the first and second natural frequencies, ω_1_ and ω_2_, with randomly distributed location parameter *c*, BEM-BEM analysis.

The expected values $E\left(\omega \right)$, coefficients of variation $\alpha \left(\omega \right)$, skewness $\beta \left(\omega \right)$, and kurtosis $\kappa \left(\omega \right)$ of the first and second natural frequencies are presented in Figure 17. A coincidence of the given three probabilistic numerical techniques is noticeable. Both obtained frequencies have apparent non-Gaussian distributions, and their statistical dispersion is rather marginal in this particular case.

The next calculations were conducted using a random fluid density, where the response functions of the first two natural frequencies were obtained in the form of the third-order polynomials:

(57) $$\omega_{1}\left(\rho_{f}\right)=59.5229924966607-0.0387879201896503\cdot \rho_{f}+\;+0.0000191378368847544\cdot {\rho_{f}}^{2}-4.08942362938761\cdot 10^{-9}\cdot {\rho_{f}}^{3}$$

(58) $$\omega_{2}\left(\rho_{f}\right)=61.8353616060936-0.0361830204214370\cdot \rho_{f}+\;+0.0000169219241151264\cdot {\rho_{f}}^{2}-3.51243115421115\cdot 10^{-9}\cdot {\rho_{f}}^{3}$$

**Figure 18:**
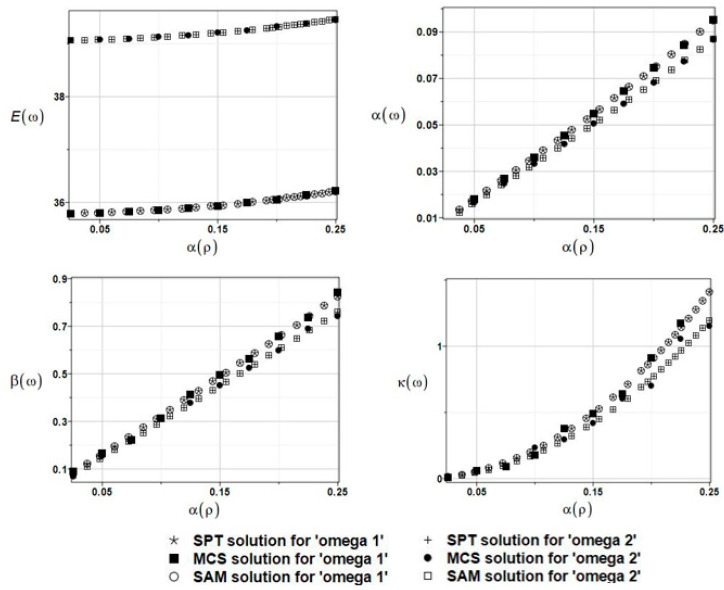
The results for the first and second natural frequencies, ω_1_ and ω_2_, with randomly distributed fluid density *ρ*_f_, BEM-BEM analysis.

The results of the calculation with the expected values $E\left(\omega \right)$, coefficients of variation $\alpha \left(\omega \right)$, skewness $\beta \left(\omega \right)$, and kurtosis $\kappa \left(\omega \right)$ of the first and second natural frequencies are presented in Figure 18. The resulting uncertainty is a little bit more influential, and these probabilistic results agree very well with those presented in Section 5.1. Probabilistic damping represents the ratio of output to input uncertainty and it equals about 3.0 here.

#### 5.2.2. Stochastic FEM-BEM Analysis

Similarly, a probabilistic analysis was performed using the random placement of the distance between the plates expressed by parameter *c,* the plate exhibiting a Gaussian uncertainty natural frequency by its mean value, and a specific range of its coefficient of variation, i.e., $\alpha \left(b\right)\in \left[0.00,0.025\right]$. The response functions of the first two natural frequencies were obtained as the third-order polynomials:

(59) $$\omega_{1}\left(c\right)=27.4360476190471+18.6499206349222\cdot c-\;-12.4809523809540\cdot c^{2}+3.05555555555603\cdot c^{3}$$

(60) $$\omega_{2}\left(c\right)=44.9528333333334-9.29686507936514\cdot c+\;+5.40476190476207\cdot c^{2}-1.19444444444455\cdot c^{3}$$

**Figure 19:**
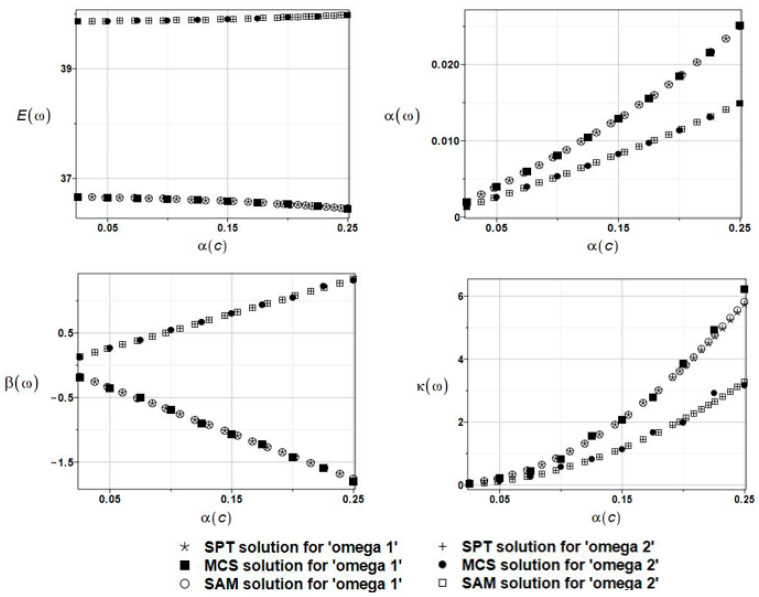
The results for the first and second natural frequencies, ω_1_ and ω_2_, with randomly distributed placement parameter *c*, FEM-BEM analysis.

The results of the calculation with the expected values $E\left(\omega \right)$, coefficients of variation $\alpha \left(\omega \right)$, skewness $\beta \left(\omega \right)$, and kurtosis $\kappa \left(\omega \right)$ of the first and the second natural frequencies are presented in Figure 19. Upon further reliability assessment, this parameter appeared to be negligible, whereas the probabilistic results coincided almost perfectly with those obtained in Section 5.2.1.

#### 5.2.3. Stochastic FDM-BEM Analysis

Similarly, the basic purpose of the third numerical experiment was to validate the proposed triple probabilistic methodology: the SAM, SPT, and MC approaches. The deterministic problem of the system of two plates immersed in fluid was solved using the FDM to describe the plate bending and the BEM to describe the occurrence of fluid (FDM-BEM analysis).

The Gaussian uncertainty natural frequency is defined by its mean value and the specific range of its coefficient of variation, i.e., $\alpha \left(b\right)\in \left[0.00,0.025\right]$. The global response functions for first two natural frequencies were obtained in this case in the form of the following third-order polynomials:

(61) $$\omega_{1}\left(c\right)=27.6376190476188+18.7613492063500\cdot c–\;-12.5166666666675\cdot c^{2}+3.05555555555581\cdot c^{3}$$

(62) $$\omega_{2}\left(c\right)=45.3350476190478-9.38817460317506\cdot c+\;+5.43452380952437\cdot c^{2}-1.19444444444468\cdot c^{3}$$

**Figure 20:**
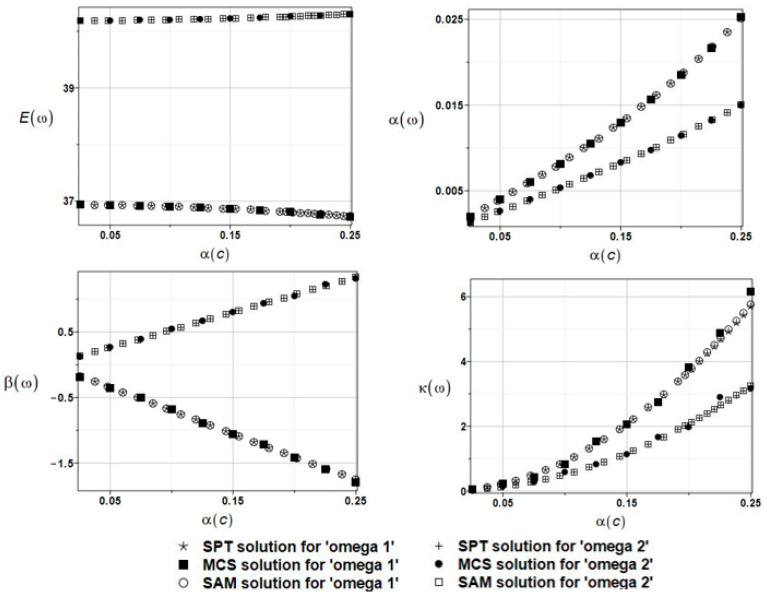
The results for the first and second natural frequencies, ω_1_ and ω_2_, with randomly distributed placement parameter *c*, FDM-BEM analysis.

The results of the calculation with the expected values $E\left(\omega \right)$, coefficients of variation $\alpha \left(\omega \right)$, skewness $\beta \left(\omega \right)$, and kurtosis $\kappa \left(\omega \right)$ of the first and second natural frequencies are presented in Figure 20.

### 5.3. Estimation of the Probabilistic Relative Entropy and the Safety Measure for Two Cantilever Plates Immersed in Fluid and Placed in One Plane along One Line

Probabilistic relative entropy *H* and the safety measure *β_H_* were estimated for the semi-analytical method (SAM) and stochastic perturbation technique (SPT), as well as the BEM-BEM, FEM-BEM, and FDM-BEM approaches and selected random parameters according to the relations (40) and (41), respectively.

#### 5.3.1. Probabilistic Entropy for the Random Parameter Describing the Distance between Plates and the BEM-BEM Approach

**Figure 21:**
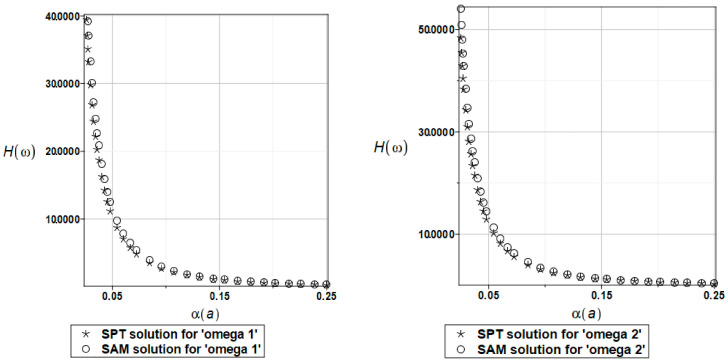
The probabilistic relative entropy with randomly distributed distance parameter *a*, BEM-BEM analysis.

The probabilistic relative entropy *H* estimated for the SAM and SPT solutions and the random distribution of the parameter *a* describing the distance between two plates for the first and second natural frequencies are presented in Figure 21.

**Figure 22:**
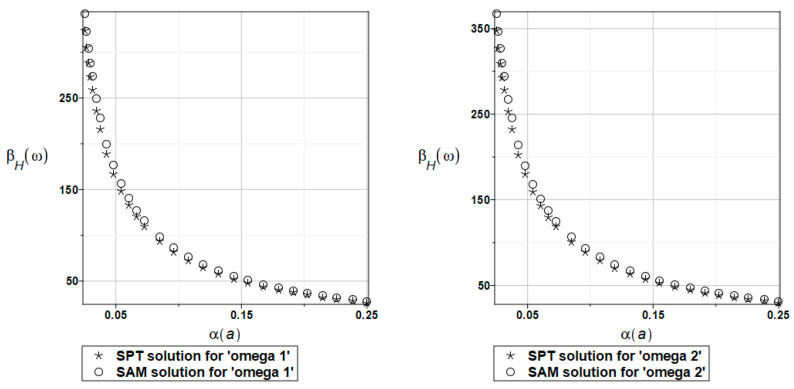
The safety measure for the first and second natural frequencies and randomly distributed distance parameter *a*, BEM-BEM analysis.

The safety measure *β_H_* estimated for the random distribution of the same parameter *a* using the SAM and SPT solutions for the first and second natural frequencies are presented in Figure 22.

It was observed that both methods returned practically the same results for the given variability range of the input uncertainty when studying the relative probabilistic entropy and its transformation to the reliability index. System safety exponentially decreases with an increase in the input CoV value; an analogous effect could be obtained while using the FORM index. A negligible effect of the given parameter uncertainty was observed in the limit values of both state functions for α(a) close to 0.25. The corresponding admissible value of the reliability index is close to 5, but equals about 40 in this example, which indicates a very small danger of resonance in this system.

#### 5.3.2. Probabilistic Entropy for the Random Fluid Density and the FEM-BEM Approach

**Figure 23:**
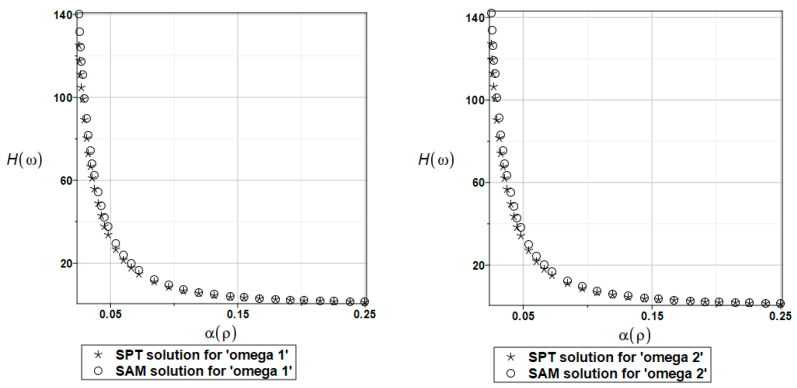
The probabilistic relative entropy for randomly distributed fluid density, FEM-BEM approach.

The probabilistic relative entropy *H* estimated for the SAM and SPT solutions with the random behavior of the fluid density for the first and second natural frequencies are presented in Figure 23.

**Figure 24:**
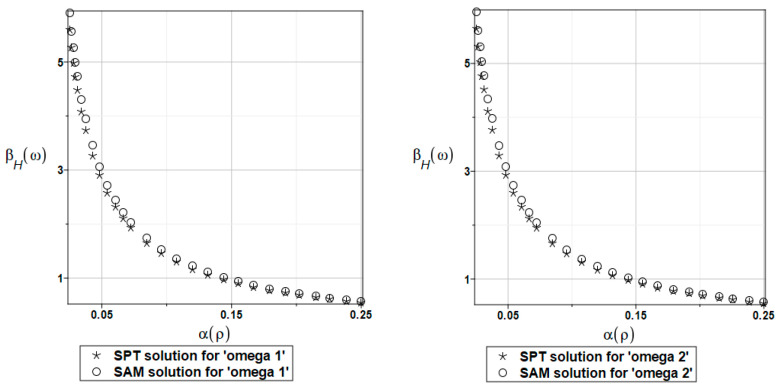
The probabilistic safety measure for randomly distributed fluid density, FEM-BEM analysis.

The safety measure *β_H_* evaluated for the random behavior of the fluid density by the SAM and SPT solutions for the first and second natural frequencies are presented in Figure 24.

In this case study, the semi-analytical and perturbation-based methodologies returned almost the same values. It is clear from these results that the plate system safety is many times smaller when randomizing the fluid density. Even for very small values of this density coefficient of variation (close to 0, which is rather unusual in experimental fluid mechanics), the reliability index based on the Bhattacharyya relative entropy is below the well-known limits presented in the literature.

#### 5.3.3. Probabilistic Entropy for the Random Fluid Density and the FDM-BEM Approach

**Figure 25:**
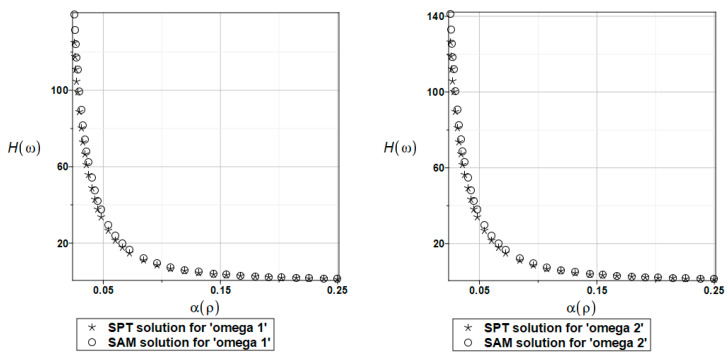
The probabilistic relative entropy for randomly distributed fluid density, FDM-BEM analysis.

The probabilistic relative entropy *H* estimated for the SAM and SPT solutions with the random behavior of the fluid density for the first and second natural frequencies are presented in Figure 25.

**Figure 26:**
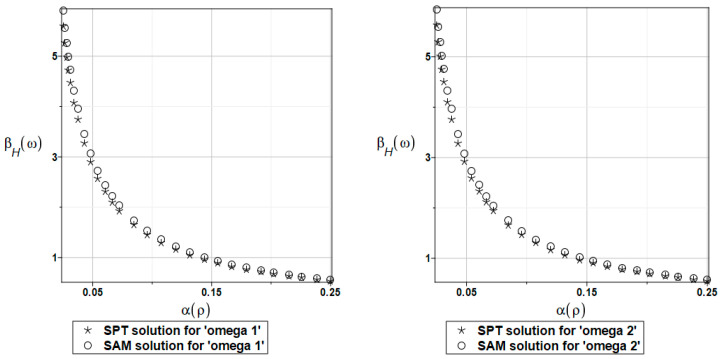
The probabilistic safety measure for randomly distributed fluid density, FDM-BEM analysis.

The safety measure *β_H_* estimated for the random behavior of the fluid density for the SAM and SPT solutions for the first and second natural frequencies are presented in Figure 26.

## 6. Concluding Remarks

The present paper presents the probabilistic approaches of Boundary Element, Finite Element, and Finite Difference Methods. Three probabilistic techniques were applied: the semi-analytical method (SAM), stochastic generalized perturbation technique (SPT), and Monte Carlo simulation (MCS).

The finite set of deterministic solutions necessary to proceed with probabilistic procedures was determined using the direct BEM approach with simplification of the formulation of the boundary and domain integral equations for isotropic and linear elastic thin (Kirchhoff–Love) plate theories. In addition, some cases were solved using the classic FEM and FDM approaches. The dynamic interaction between a plate and fluid was specified by the theory of the potential of the double layer for a fluid and the direct BEM approach.

From the viewpoint of the number of computational operations necessary to solve a single deterministic task, the optimal choice is the use of the FEM-BEM approach. In turn, the most regular arrangement of plate boundary elements and subsurfaces is only possible in terms of the BEM-BEM. In the other two cases (FEM-BEM and FDM-BEM), in order to maintain identical dimensions of the finite elements or the grid of points, the location of subsurfaces for liquids should always be adjusted so that the vertical component of the inertial force vector of the liquid coincides with the vertical component of the inertial force vector of the plate itself. Each of the approaches (BEM-BEM, FEM-BEM, and FDM-BEM) used requires the construction of a complete and generally asymmetric matrix **H**. This matrix must then be inverted, which, with low accuracy, may cause numerical errors. Because of the large number of internal subsurfaces into which both surfaces of the plates are divided, it is time-consuming. From the viewpoint of the simplest formulation of the presented problem, the BEM-BEM approach seems to be the simplest and most useful. According to this approach, the division of the interior of the plates into subareas related to the liquid was the most regular and simple.

One can even be tempted to say that the joint implementation and application of SBEM, SFEM, and SFDM are not available in the literature in terms of any of the known stochastic methods; in this study, three approaches are used simultaneously. Third-degree polynomials were applied to fit the response signal curves for random parameters such as fluid density, plate placement, plate thickness, and collocation point location. It must be mentioned that the time consumption of both the semi-analytical and stochastic perturbation approaches are quasi-similar. The most time-consuming is the Monte Carlo simulation technique, which is attributable to the number of trials. The fluid density and boundary element collocation point placement (for BEM-BEM analysis) randomness seem to be negligible. From the viewpoint of the use of reliability measures expressed best by the coefficient *β_H_*, one can observe that their values drop sharply when the value of the argument, the uncertainty coefficient, increases.

The simplest of the random formulations—the SAM approach—allows the obtention of random moments in analytical forms, which is very convenient for further computational implementation in any academic and commercial BEM, FEM, or FDM programs.

The presented concept can also be successfully applied to the analysis of the dynamics of the control surfaces of ships or aircraft.

## Figures and Tables

**Table 1:** The influence of the parameter $\tilde{\delta }$ on the successive natural frequencies for a single cantilever plate fully immersed in water.

Natural Frequencies *ω* [rad/s]								
BEM-BEM—Present			Anal. Solution [44]		FEM-BEM [48]		FDM-BEM [48]	
$\tilde{\delta }$	*ω*_1_	*ω*_2_	*ω*_1_	*ω*_2_	*ω*_1_	*ω*_2_	*ω*_1_	*ω*_2_
0.001	37.582	105.379	34.9110	114.185	38.716	108.985	38.146	117.352
0.01	37.540	105.262						
0.02	37.536	105.340						

**Table 2:** Influence of the parameter $\tilde{\delta }$ on the successive natural frequencies for the system of two cantilever plates fully immersed in water and *a* = 1.0 m.

Natural Frequencies *ω* [Rad/s], BEM-BEM—Present						
$\tilde{\delta }$ [m]	0.0001	0.00025	0.0005	0.001	0.005	0.025
*ω*_1_	37.403	37.401	37.398	37.393	37.353	37.346
*ω*_2_	37.731	37.730	37.727	37.721	37.681	37.674
*ω*_3_	105.187	105.185	105.171	105.151	105.026	105.108
*ω*_4_	105.427	105.425	105.411	105.391	105.266	105.347

**Table 3:** Natural frequencies for the system of two cantilever plates fully immersed in water and *a* = 1.0 m obtained for the FEM-BEM and FDM-BEM approaches.

Natural Frequencies *ω* [Rad/s]							
FEM-BEM—Present				FDM-BEM—Present			
*ω*_1_	*ω*_2_	*ω*_3_	*ω*_4_	*ω*_1_	*ω*_2_	*ω*_3_	*ω*_4_
38.238	38.552	107.693	107.916	38.537	38.854	116.047	116.295

**Table 4:** The comparison of the results obtained from the system of two cantilever plates fully immersed in water and *a* = 1.0 m under the FEM-BEM approach and three different FEM and BEM meshes.

Plate FEM mesh	11×11	16×16	21×21
Fluid BEM mesh	10×10	15×15	20×20
$\omega_{1}$	37.567	38.238	38.593
$\omega_{2}$	37.896	38.552	38.898
$\omega_{3}$	105.684	107.693	108.786
$\omega_{4}$	105.924	107.916	109.000

**Table 5:** The comparison of the results obtained from the system of two cantilever plates fully immersed in water and *a* =1.0 m under the FDM-BEM approach, three different FDM grids, and BEM meshes.

Plate FDM grid	10×10	20×20	30×30
Fluid BEM mesh	5×5	10×10	15×15
$\omega_{1}$	36.557	37.979	38.537
$\omega_{2}$	36.939	38.312	38.854
$\omega_{3}$	121.223	117.230	116.047
$\omega_{4}$	121.586	117.509	116.295

**Table 6:** An influence of the parameter $\tilde{\delta }$ on the natural frequency *ω* for the system of two cantilever plates completely immersed in water and *a* = 1.0 m.

Natural Frequencies *ω* [Rad/s] BEM-BEM—Present						
$\tilde{\delta }$ [m]	0.0001	0.00025	0.0005	0.001	0.005	0.025
*ω*_1_	35.792	35.790	35.787	35.781	35.743	35.736
*ω*_2_	39.071	39.069	39.066	39.059	39.018	39.012
*ω*_3_	103.723	103.721	103.707	103.687	103.564	103.644
*ω*_4_	106.788	106.286	106.771	106.751	106.624	106.707

**Table 7:** Natural frequencies *ω* for the system of two cantilever plates fully immersed in water and *c* = 1.0 m obtained for FEM-BEM and FDM-BEM approaches.

Natural Frequencies *ω* [Rad/s]							
FEM-BEM—Present				FDM-BEM—Present			
*ω*_1_	*ω*_2_	*ω*_3_	*ω*_4_	*ω*_1_	*ω*_2_	*ω*_3_	*ω*_4_
36.658	39.867	106.292	109.219	36.935	40.188	114.487	117.746

**Table 8:** The comparison of the results from the system of two cantilever plates fully immersed in water, *c* = 1.0 m obtained using the FEM-BEM approach, and three different FEM and BEM meshes.

Plate FEM mesh	11×11	16×16	21×21
Fluid BEM mesh	10×10	15×15	20×20
$\omega_{1}$	35.950	36.658	37.029
$\omega_{2}$	39.238	39.867	40.201
$\omega_{3}$	104.218	106.292	107.417
$\omega_{4}$	107.286	109.219	110.274

**Table 9:** The comparison of the results from system of two cantilever plates fully immersed in water, *c* = 1.0 m obtained using the FDM-BEM approach, and three different FDM grids and BEM meshes.

Plate FDM grid	10×10	20×20	30×30
Fluid BEM mesh	5×5	10×10	15×15
$\omega_{1}$	34.755	36.332	36.935
$\omega_{2}$	38.429	39.682	40.188
$\omega_{3}$	119.050	115.530	114.487
$\omega_{4}$	123.599	119.089	117.746

## Data Availability

The data can be obtained from the Authors.
